# Sustained Administration of Hormones Exploiting Nanoconfined Diffusion through Nanochannel Membranes

**DOI:** 10.3390/ma8085241

**Published:** 2015-08-13

**Authors:** Thomas Geninatti, R. Lyle Hood, Giacomo Bruno, Priya Jain, Eugenia Nicolov, Arturas Ziemys, Alessandro Grattoni

**Affiliations:** 1 Nanomedicine Department, Houston Methodist Research Institute, Houston, TX 77030, USA; E-Mails: rlhood@houstonmethodist.org (R.L.H.); pjain@houstomethodist.org (P.J.); enicolov@houstonmethodist.org (E.N.); aziemys@houstonmethodist.org (A.Z.); 2 College of Materials Science and Engineering, University of Chinese Academy of Sciences, Beijing 100049, China; E-Mail: tgeninatti2@houstonmethodist.org; 3 Electronics and Telecommunications Department, Politecnico di Torino, Turin 10024, Italy; E-Mail: gbruno2@houstonmethodist.org

**Keywords:** nanochannel membrane, nanoconfinement, nanoscale diffusion, drug delivery, hormone replacement

## Abstract

Implantable devices may provide a superior means for hormone delivery through maintaining serum levels within target therapeutic windows. Zero-order administration has been shown to reach an equilibrium with metabolic clearance, resulting in a constant serum concentration and bioavailability of released hormones. By exploiting surface-to-molecule interaction within nanochannel membranes, it is possible to achieve a long-term, constant diffusive release of agents from implantable reservoirs. In this study, we sought to demonstrate the controlled release of model hormones from a novel nanochannel system. We investigated the delivery of hormones through our nanochannel membrane over a period of 40 days. Levothyroxine, osteocalcin and testosterone were selected as representative hormones based on their different molecular properties and structures. The release mechanisms and transport behaviors of these hormones within 3, 5 and 40 nm channels were characterized. Results further supported the suitability of the nanochannels for sustained administration from implantable platforms.

## 1. Introduction

Unbalanced natural hormone production is a pervasive condition typically treated with hormone replacement therapy (HRT) [1,2,3]. HRT is usually performed through a series of oral tablets or injections to mimic physiological cycles, as each administration results in a rapid increase and decrease of the blood serum concentration levels. However, these wide fluctuations do not accurately mimic healthy physiological hormone availabilities [4]. Furthermore, treatment outcomes are heavily dependent on patient compliance to prescribed regimens [5]. Long-term HRT is used to treat a variety of diseases, including renal failure, cardiovascular diseases, and hormonal contraception [6,7,8,9]. The protection of these agents from physiological interactions is crucial for maintaining stability and treatment efficacy [10]. To address these needs within the field of hormone delivery, several groups have developed sustained administration approaches capable of maintaining more consistent serum concentrations than traditional methods while reducing the total number of treatments given [11,12]. Several systems have been developed, including long-acting injections, biodegradable polymers, and conjugated nanocarriers [13,14,15,16,17]. However, studies have shown that these approaches suffer from inconsistent dosage maintenance [5]. For example, release from biodegradable polymers has been demonstrated to lead to a front-loaded, exponential delivery rate based on the diminishing surface area of the degrading implant [18].

These shortcomings, when paired with the particular needs of many HRT regimens requiring life-long treatment, motivate the development of novel drug delivery strategies. Our research group developed a nanofluidic membrane providing controlled release of therapeutics through physically and electrostatically constraining molecular diffusion at the nanoscale [19,20,21]. Constant, zero-order release is achieved by tailoring the height of the nanofluidic channels to near the hydrodynamic radii of the diffusing molecule of interest [22,23,24,25,26]. No pumping mechanisms or valves are required to drive transport through the membrane structure, as the system relies on the concentration gradients of diffusing molecules between an inner reservoir and the external environment. Therapeutic proteins, such as hormones, present unique and interesting characteristics influencing their transport under nanoconfinement conditions [27]. As sustained and controlled delivery of hormones addresses a current clinical need, this study focuses on the characterization of hormone transport across the nanochannel system. The nanofluidic membranes do not mimic natural hormone secretion, which occur at different levels throughout the day. However it has been shown that, constant delivery is clinically acceptable and more desirable than multiple bolus administrations [22,28].

In this work, we hypothesize that long-term, zero-order hormone release can be achieved through the use of our novel drug delivery platform. To test this hypothesis, the nanoconfined diffusive transport of three model hormones was studied within nanochannel membranes. These hormones were selected based on their size, charge, and distribution ratio (logD): levothyroxine, osteocalcin, and testosterone. Experiments leveraged silicon membranes incorporating 349448 identical and parallel nanochannels. Different membranes with nanochannels heights ranging from 3 to 40 nm were used to evaluate diffusive transport. Further analysis of the transport behavior and intrachannel distribution of these model hormones enabled evaluation of this nanochannel platform’s utilization for clinical hormone administration. 

## 2. Results and Discussion

Based on physicochemical properties of the three agents, nanofluidic membranes possessing negative surface charge and nanochannel heights including 3, 5 and 40 nm were employed to study their transport mechanisms in the context of controlled release.

### 2.1. Levothyroxine

**Figure 1:**
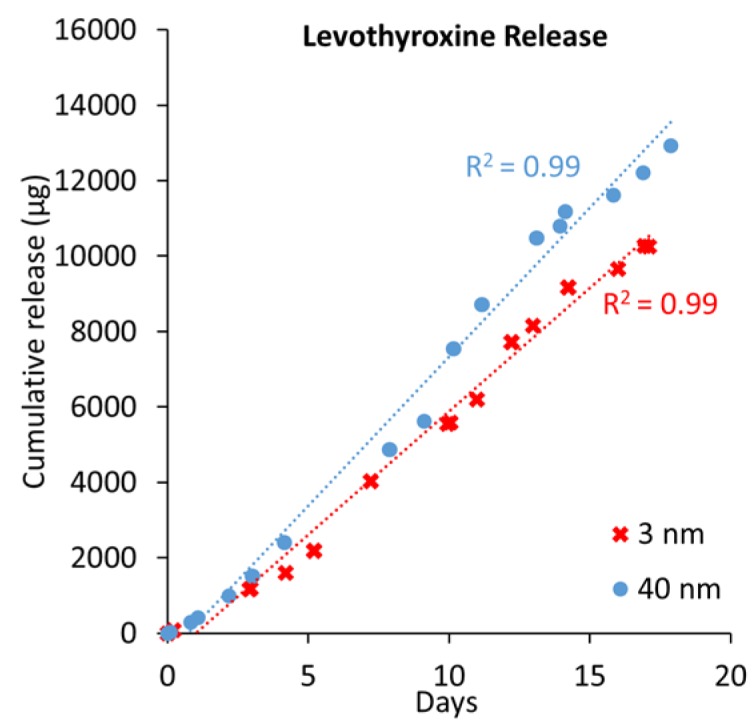
Levothyroxine cumulative release from 3 and 40 nm nanochannel membranes and their linear fits (blue and red dotted lines).

Levothyroxine is a small, negatively charged (−0.5*e* at pH 7.4, Table 1), and hydrophobic molecule (logD 1.76, Table 1) used to treat several forms of hypothyroidism [29,30,31]. Levothyroxine was released from implantable capsules maintained under simulated physiological conditions. The cumulative release curves for levothyroxine are shown in Figure 1.

**Table 1:** Properties of released molecules. LogD: distribution ratio.

Properties	Osteocalcin	Levothyroxine	Testosterone
Mass (Da)	5929	777	288
Net Charge (pH 7.4)	−6	−0.5	0
Radius (Å)	10	4.3	4.1
LogD (PH 7.4)	~−6	1.76	3.16

**Figure 2:**
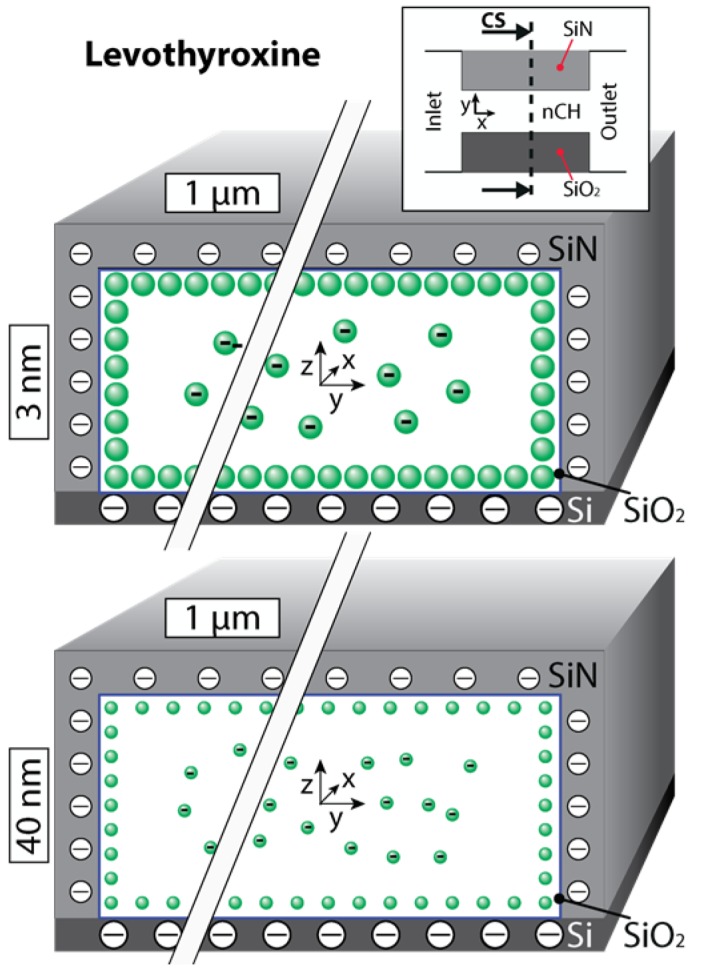
Nanochannel cross (CS) sections depicting levothyroxine distributions within 3 and 40 nm nanochannel membranes are shown. Neutrally charged molecules move near the wall surface while negatively charged molecules tend to migrate towards the center of the nanochannel. Please note that the aspect ratio was lessened for better visual representation of the molecular distribution.

Spatial confinement by the nanochannels seemed to linearize levothyroxine’s diffusive transport (Figure 2). This linearization may have been more attributable to direct spatial confinement than to ionic redistribution within the channel [23,32]. Due to a nearly equivalent presence of two stable levothyroxine configurations, neutral and negatively charged (−1*e*), the overall charge is approximately −0.5*e* (Table 1). Therefore, electrostatic interactions affect about half of the population, leading to an accumulation of charged species towards the channel center, a distribution previously termed gated diffusion (GD). The behavior of the neutral molecules would most likely be attributable to their positive logD, as water interaction minimization would lead to aggregation at the nanochannel surfaces (ANS). Leveraging these experimental values, it was possible to develop an empirical approximation of the flux contributions between the GD and ANS for the smallest nanochannels as:

GD = *J* − ANS and ANS = *D* × GD (1)

where *J* is the diffusion rate of levothyroxine, and *D* is the distribution ratio calculated from logD. Assuming a proportional correlation between the area of the nanochannel, the bulk diffusion and ANS being constant, it is possible to normalize the diffusive rate (GDN) from the surface (*A*) as:

*J* = ANS + GD = ANS + GDN × *A* (2)

Equation (2) can decouple the role of the bulk diffusion from the near-surface diffusion. By incorporating the value for diffusive rate determined from the release through 3 nm nanochannels, it is possible to predict the release rate for the 40 nm nanochannels. The experimental value is comparable, within 10% of the prediction, additionally supporting the hypothesis of both phenomena being present. An alternative explanation for the similar release is attributable to the consistent inlet and outlet microchannel network for all membranes tested. These may present a significant contribution to the overall effective “diffusive resistance” of the system and reduce the contribution of nanochannels in determining the overall membrane’s release rate.

### 2.2. Osteocalcin

Osteocalcin is a large, negatively charged, osteoclast-derived hormone that is most abundant in the noncollagenous protein of bone matrix. Studies have revealed it to be a multifunctional hormone that promotes insulin secretion and testosterone biosynthesis, among other activities [33]. A low LogD value (~−6, Table 1) at neutral pH renders osteocalcin very hydrophilic and homogeneously distributed in bulk solution. Osteocalcin was released from implantable capsules under simulated physiological conditions, sampling its release for 10 days. Samples were analyzed with a micro-bicinchoninic acid (BCA) protein assay kit.

**Figure 3:**
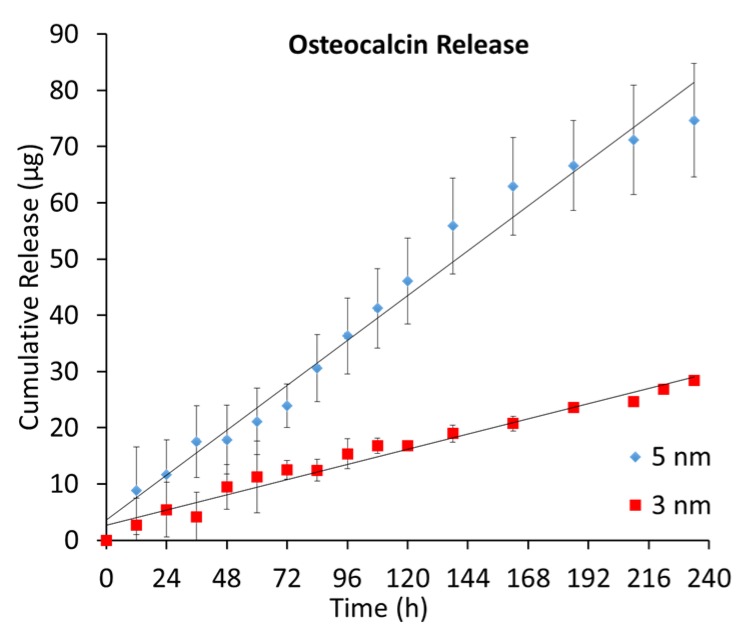
Average cumulative release of osteocalcin from 3 and 5 nm nanochannel membranes (*n* = 3). Experimental points were collected every 12 h for 10 days.

**Figure 4:**
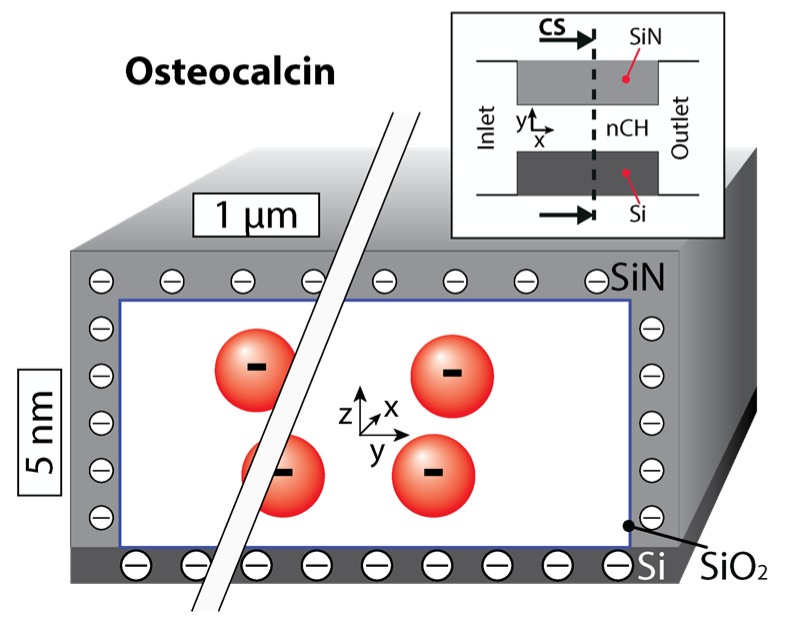
Cross-section of a nanochannel qualitatively showing the distribution of a 5 nm nanochannel. Please note that the aspect ratio was lessened for better visual representation of the molecular distribution.

The release behavior (Figure 3) is consistent with the zero-order release trend observed in levothyroxine and previous studies for negative transport under nanoconfinement [23,34]. However, it demonstrated a much greater dependence on nanochannel size than leveothyroxine. Osteocalcin molecules are repelled from the negative surfaces of the nanochannel, leading to high concentrations in the center that decrease towards the walls. This is exhibited in Figure 4, which provides a qualitative estimate of osteocalcin redistribution through a cross-section of the channel. Over the 10 day period, approximately 78% of the loaded hormone released from the 5 nm membranes, while only 28% released from the 3 nm. There was also a significant difference in release rate, as the osteocalcin transport through the 5 nm membranes was approximately 3 times the rate through the 3 nm. This difference cannot be explained by the previous Equations (1) and (2) describing gated diffusion. We speculate that this behavior can be primarily attributed to the tight spatial confinement of diffusing molecules provided by the nanochannels. As osteocalcin has a relatively large molecular weight (5929 Da, Table 1) and hydrodynamic radius (1.0 nm), we suggest this transport to be defined by the ratio between nanochannel height and molecular diameter, which approaches unity for the 3 nm case with our innovative, nanofluidic membrane. These results are highly interesting and motivates additional questions on whether the platform can be leveraged for filtering and sorting applications. Since osteocalcin molecules express high negative charge (−6*e*), they lend themselves to the enhanced release control attainable with our next generation nanochannel membranes, which incorporate platinum electrodes to provide an overt, additional electrostatic potential for ion manipulation [35,36,37].

### 2.3. Testosterone

**Figure 5:**
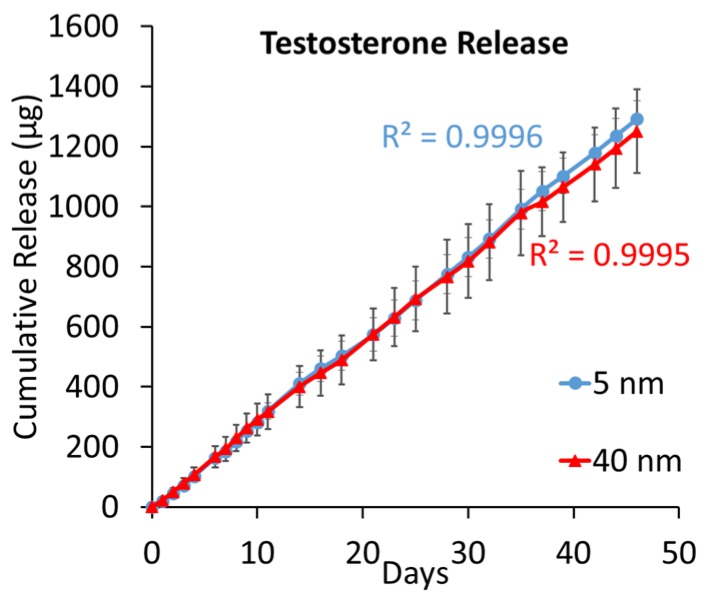
Average cumulative release of testosterone for both 5 (blue) and 40 (red) nm nanochannel membranes. The grey lines highlight the standard deviation. Theoretical fitting value is report for both releases.

Testosterone is a small molecule of comparable size and charge with levothyroxine (4.1 Å of radius and neutral charge, Table 1), but with substantially lower mass (288 Da, Table 1). The standard clinical intervention for low natural secretion of testosterone is frequent injections for replacement. Similar to osteocalcin and levothyroxine, testosterone was released from implantable capsules under simulated physiological conditions. The cumulative release of testosterone is exhibited in Figure 5.

**Figure 6:**
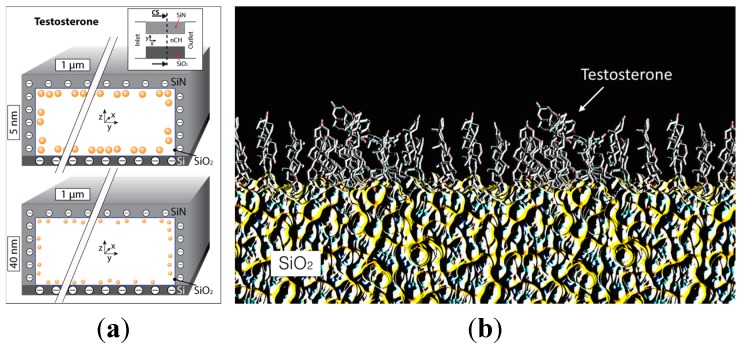
(**a**) Cross section of a nanochannel highlighting the testosterone distribution during its release across 5 and 40 nm nanochannels membranes. Please note that the aspect ratio was lessened for better visual representation of the molecular distribution; (**b**) Testosterone single monolayer on a silica (SiO_2_) surface.

This molecule is approximately a billion times more hydrophobic than osteocalcin and 25 times more than levothyroxine. Testosterone’s high hydrophobicity and lack of charge would likely cause molecules to separate from the aqueous solvent to aggregate at the hydrophobic silica surfaces [38], forming two different and immiscible phases. The results plotted in Figure 5 clearly demonstrate a comparable release from 5 and 40 nm nanochannel membranes. A parallel computational study analyzed the testosterone properties in aqueous solution both with and without the presence of nanochannels. Testosterone aggregates under aqueous conditions, hiding the hydrophobic carbon cores and exposing oxygen terminals to reduce its thermodynamic energy [39]. These configurations were found to be very stable, which would prevent molecule aggregation. In addition, the mass ratio found between the surface monolayer in the nanochannel and the bulk volume is 2% for a 5 nm nanochannel and 20% for a 40 nm nanochannel (surface absorption was neglected in this computational model), as represented in Figure 6a. In order to increase the release rate of testosterone, two approaches may be adopted: employ nanochannels with a height significantly larger (≥200 nm) to permit bulk diffusion at the channel’s center or increase the number of nanochannels.

## 3. Materials and Methods

### 3.1. Nanochannel Membrane Fabrication

**Figure 7:**
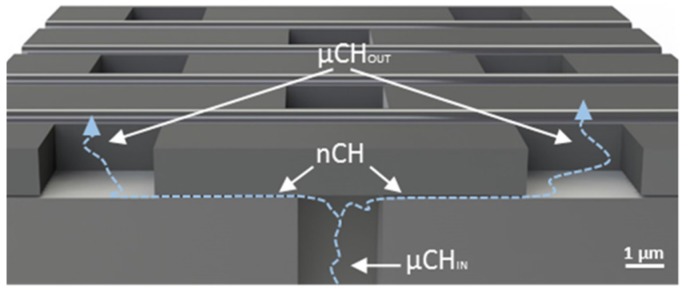
Cross section of the nanofluidic membrane. The membranes used in the manuscript have nanochannels with 3 μm width and 3 μm length. The microchannels (μCh_IN_) and outlet microchannels (μCh_OUT_) were 30 and 1.7 μm in length, respectively. The nanochannel (nCH) employed presented height ranging from 3 to 40 nm.

Structure and fabrication protocols for manufacturing nanochannel membranes have been presented previously [40,41]. Briefly, the membranes were 6 × 6 mm wide and 730 μm in height (Figure 7). To clearly present the structure of the membrane, a cross-section has been shown in Figure 7. The membrane presented 161 macrochannels (MCh), each 200 × 200 μm wide and 670 μm height. Every MCh contained 38 × 37 (1406) inlet microchannels (μCh_IN_), each 3 × 3 μm wide and 30 μm in height. Every μCh_IN_ ended in two nanochannels, each being 3 μm in length and width, and the nominal number of nm in height. The nanochannels were connected to outlet microchannels (μCh_OUT_) 3 × 3 μm wide and 1.7 μm height. To develop nanochannel membranes for hormone delivery, nanochannels sizes of 3, 5 and 40 nm were utilized. The total number of nanochannels in a membrane is 349,448.

### 3.2. Levothyroxine Release

Levothyroxine (Sigma-Aldrich Inc., St. Louis, MO, USA) release tests were performed employing fully assembled and loaded nanochannel capsules [22]. 3 nm (*n* = 1) and 40 nm (*n* = 1) membranes were used. Two capsules with integrated membranes expressing nanochannels 3 nm in height and a third with 40 nm channels were loaded with approximately 900 μL of 20 mg/mL levothyroxine solution. For the release experiment, each capsule was immersed in a borosilicate glass bottle containing 50 mL of Millipore water. Samples were taken daily with replacement. Constant homogenization of the sink solution was performed by magnetic stirring at 300 rpm. Absorbance measurements of the samples were taken with a UV/Vis spectrophotometer (Beckman Coulter, Inc., DU 730, Brea, CA, USA) at a wavelength of 240 nm. Data were normalized with respect to absorbance at *t* = 0, and the cumulative release of the agents were obtained through comparison to a standard curve. 

### 3.3. Osteocalcin Release

Osteocalcin (AnaSpec Inc., Fremont, CA, USA) release was performed with similar nanochannel capsules as above [22]. The reservoir was loaded with 250 μL of osteocalcin solution at a concentration of 400 μg/mL in phosphate-buffered saline (PBS). 3 and 5 nm (*n* = 3 each) nanochannel membranes were used. Each capsule was immersed in a borosilicate glass bottle containing 5 mL of Millipore water. 500 μL of samples were taken every 12 h for 10 days. The amount of sample removed was replaced each time with the buffer solution. Hormone concentration was determined with a micro BCA protein assay kit (Life Technologies, Carlsbad, CA, USA) using bovine serum albumin as a standard.

### 3.4. Testosterone Release

Testosterone (Sigma-Aldrich Inc.) release tests were also performed with nanochannel capsules. Membranes with 5 nm (*n* = 3) and 40 nm (*n* = 3) nanochannels were employed. Each capsule was loaded with a testosterone solution of 2.5 mg/mL in aqueous solvent. For the release experiment, each capsule was immersed in a borosilicate glass bottle containing 50 mL of Millipore water. Samples were taken daily with replacement. Constant homogenization of the sink solution was performed by magnetic stirring at 300 rpm. Absorbance measurements of the samples were taken with a UV/Vis spectrophotometer at a wavelength of 241 nm. Data were normalized with respect to absorbance at *t* = 0, and the cumulative release of the agents were obtained through comparison to a standard curve. 

### 3.5. Computational Methods

Molecular dynamic simulations were carried out in analogous fashion as in Ziemys *et al.* [32,42,43]. Briefly, the simulations were carried out using NAMD 2.6 [44] with a TIP3P water model [45] and NVT ensembles. All molecules involved in the model were simulated with the CHARMM22 force. The silica 5 nm nanochannel model was prepared as described by Cruz-Chu and colleagues [46]. The whole model size was with dimensions 4 × 4 × 8 nm. The system was fully filled with water and ions to balance the total charge. Thirty six testosterone molecules were dissolved. Periodic boundary conditions were applied in all directions. The whole model was minimized, equilibrated and later production simulation executed over 120 ns using 2fs integration step.

## 4. Conclusions

Nanochannel membranes successfully demonstrated sustained *in vitro* delivery of three clinically-relevant hormones: levothyroxine, osteocalcin, and testosterone. These model molecules were chosen as they have diffusion-relevant characteristics representative of a broad range of hormones used in HRT. Release over different timescales proved the system’s flexibility and capability to provide sustained release despite different molecular weights, charges, and distribution ratios. In the case of the negatively charged, hydrophobic molecule levothyroxine, a zero-order release was achieved, and release rate directly correlated to the channel size. This was attributed to the dual contributions of electrostatic interactions and aggregation at the nanochannels’ surfaces. The linear release of osteocalcin was attributed to the electrostatic interaction between the ions and the walls of the 5 nm nanochannels, while high physical confinement substantially reduced release in the 3 nm case. Testosterone, a neutral and highly hydrophobic molecule, exhibited a zero-order release profile independent from nanochannel height (5 and 40 nm presented the same cumulative release). This was ascribed to the hydrophobic tendency of testosterone to aggregate in clusters and along the nanochannel surfaces, limiting diffusion across the membrane. These results highlighted the flexibility of the nanochannel platform, supporting its potential employment for long-term hormone replacement and other therapeutic approaches requiring sustained release.
